# The association between Children's sleep quality and juvenile idiopathic arthritis

**DOI:** 10.3389/fped.2025.1703948

**Published:** 2026-01-05

**Authors:** Linxia Li, Yazhen Di, Zhanli Liu, Zelin Hao

**Affiliations:** 1 Department of Neurology, Hangzhou Children’s Hospital, Hangzhou City, Zhejiang Province, China; 2 Department of pediatrics, Ningbo Women and Children’s Hospital, Ningbo City, Zhejiang Province, China; 3 Department of Neurosurgery, The Affiliated Hospital of Hangzhou Normal University, Hangzhou City, Zhejiang Province, China

**Keywords:** functional status, juvenile idiopathic arthritis, pediatrics, qualityof sleep, sleep disturbance

## Abstract

**Objective:**

To investigate possible sleep disturbances in children with juvenile idiopathic arthritis (JIA).

**Methods:**

This case-control investigation evaluated 48 JIA patients vs. 90 healthy controls through comprehensive clinical assessments. Disease activity was quantified using the Juvenile Arthritis Disease Activity Score (JADAS-27), while health-related quality of life was measured with the Child Health Questionnaire (CHQ-PF28). Pain intensity was assessed through dual Methods: Visual Analog Scale (VAS) and Wong Baker Faces Scale. Fatigue levels were evaluated via the Pediatric Quality of Life Inventory Multidimensional Fatigue Scale (PedsQL-MFS), and sleep quality was analyzed using both the Children's Sleep Habits Questionnaire (CSHQ) and Sleep Disturbance Scale for Children (SDSC). All statistical analyses were conducted in SPSS 26.0, employing independent *t*-tests for intergroup comparisons and Spearman correlation analysis for variable relationships.

**Results:**

The incidence of sleep disturbance was significantly higher in the JIA group (*P* < 0.01). CSHQ assessments reveal abnormal total scores and six subdomains (sleep duration, daytime sleepiness, etc.; all *P* < 0.05), with specific correlations: total scores link to disease activity (JADAS-27/AJC; *P* = 0.021–0.05), shortened sleep duration strongly associates with ESR/functional status (*P* = 0.002/0.006), and daytime sleepiness connects to physician global assessments (PGA/PGE; *P* = 0.017–0.029). SDSC evaluations indicate abnormalities in four domains (all *P* < 0.05), notably: night hyperhidrosis correlates with disease activity markers (*P* = 0.032–0.039), excessive sleepiness associates with fatigue/functional impairment (*P* = 0.014/0.038), and arousal disorders relate to medication use (*P* = 0.029).

**Conclusions:**

JIA patients show significantly higher rates of sleep disorders compared to healthy children, primarily due to disease activity, functional impairment, and medication effects.

## Highlights

- Revealed exceptionally high sleep disturbance prevalence in juvenile idiopathic arthritis (JIA) patients (85% in children <11 years; 28.6% in adolescents), significantly exceeding healthy controls (*P* < 0.01).
- Identified medication-driven sleep disruptions: Polypharmacy (≥3 drugs) in 42.9% of JIA adolescents strongly associated with arousal disorders (*P* = 0.029) and night hyperhidrosis (*P* = 0.046).
- Established disease activity-sleep linkage: JADAS-27 scores correlated with CSHQ total scores (*P* = 0.05) and SDSC night sweats (*P* = 0.032–0.039), confirming inflammation's role in sleep pathology.
- For age-stratified sleep assessment, we used CSHQ (<11 years) and SDSC (≥11 years), both validated in pediatric populations and shown to improve assessment accuracy compared to single-tool approaches (1, 2).

## Introduction

Juvenile idiopathic arthritis (JIA) is one of the most common rheumatic diseases in children, characterized by a group of heterogeneous conditions that typically begin before the age of 16, with a duration of at least 6 weeks, and excluding other infectious or malignant diseases (3). JIA has an estimated prevalence of 40 case per 100,000 children, predominantly affecting girls aged 1–3 years, and boys aged 8–10 years (2). JIA is primarily marked by chronic synovitis and can lead to systemic organ dysfunction, making it a significant cause of disability and blindness in childhood. In 2019, the Pediatric Rheumatology International Trials Organization (PRINTO) defined JIA as an inflammatory disease that occurs before the age of 18, with inflammation lasting at least 6 weeks (3). Treatment options for JIA include nonsteroidal anti-inflammatory drugs (4) (NSAIDs), disease-modifying antirheumatic drugs (5) (DMARDs), and biologics (6, 7). With the active treatment of patients and the continuous improvement in prognosis, higher demands are now placed on the quality of life for children with JIA, particularly in terms of sleep. Currently research provides limited evidence regarding sleep patterns and their determinants in juvenile idiopathic arthritis (JIA) patients. The degree of daytime drowsiness is linked to the assessment of disease activity and pain by doctors and parents, as well as the impact of JIA on children's daily lives. This study seeks to investigate the sleep disturbances in pediatric patients with JIA and their potential associations with fatigue levels, physical functional limitations, disease activity indices, and pain severity.

## Patients and methods

### Participants

From June to December 2021, 48 patients aged 4–18 years, who were admitted to the Department of Pediatric Rheumatology and Immunology at Ningbo Women and Children's Hospital and met the inclusion and exclusion criteria, were diagnosed according to the classification standards of the International League of Associations for Rheumatology (ILAR, 2004 revision) or the new JIA classification proposed by the 2018 International Trial Organization for Pediatric Rheumatology (PRINTO). A control group of 90 children, matched in age and gender and without chronic diseases, was selected from those hospitalized at Ningbo Women and Children's Hospital during the same period. Exclusion criteria included: (1) having a second rheumatic disease or other chronic diseases; (2) having mental defects or psychological issues; (3) using drugs that affect sleep (except for those required for arthritis treatment); (4) having tumors, injuries, severe infections, or other underlying conditions; (5) being over 18 years old; (6) the child's guardian not understanding the questionnaire or refusing to participate in the study. A total of 130 potential parent-child dyads in the control group were approached during the study period. Of these, 90 child agreed to participate, yielding a participation rate of 69.2%. This information is now included to allow for a clearer evaluation of potential selection bias. All children and their parents were informed of the research purpose and provided with an informed consent form. The study was approved by the Research Ethics Committee of Ningbo Women and Children's Hospital (Approval Number: EC2021-037).

Collect general information of hospitalized children, including demographic data such as name, sex, age, date of birth, etc., and summarize the medical records of JIA patients to collect the following information: JIA course, drugs taken during the study visit, painful joints, swollen joints, erythrocyte sedimentation rate, RF, MAGNETIC resonance report, adverse factors for prognosis, etc.

### JIA scale of related variables

In the JIA group, we assessed disease activity using the Juvenile Arthritis Disease Activity Score with 27 joints (8) (JADAS-27). This includes the Active Joint Count (AJC), Physician's Global Assessment (PGA), Parental Global Evaluation (PGE), and erythrocyte sedimentation rate (ESR). The assessments by doctors and parents were based on a 100 mm VAS scale, where 0 indicates no disease activity and 100 indicates very severe disease. After collecting blood samples to measure ESR, the following conversion is used: [ESR (mm/h) −20]/10, which converts ESR values <20 mm/h to 20 and ESR values > 120 mm/h to 120 (15). The JADAS-27 is the sum of these four variables.

The Childhood Health Questionnaire Parent Form 28 (CHQ-PF28) is used to assess the functional status of children with idiopathic arthritis. It covers 13 areas, including physical activity, academic performance, social activities, physical condition, behavior, emotional state, life satisfaction, and its impact on family members. Each question is rated on a scale from 0 to 4, and the total score is calculated by summing the scores of each question. A lower total score indicates poorer health-related quality of life and more pain and overall health issues.

Pain is assessed using the Visual Analog Scale (VAS), the line-drawing method, or the Wong-Baker pain intensity rating. For the VAS line-drawing method, draw a long line (100 mm) between no pain and severe pain, without any marks, numbers, or words to avoid influencing the assessment results. One end represents no pain, and the other end represents severe pain. Have the child draw a cross at the point on the line that best reflects their pain level. If the child is too young or if it is difficult to use the aforementioned methods for pain assessment, a scoring system with pictures of different facial expressions can be used (Wong-Baker pain intensity rating): no pain, a little pain, slightly painful, more painful, very painful, most painful (0 points, 2 points, 4 points, 6 points, 8 points, 10 points).

Fatigue is assessed using the PedsQLTM Multidimensional Fatigue Scale (PedsQLTM MFS). This 18-item scale is an effective and reliable tool for measuring symptom-specific fatigue in patients with JIA. It includes questions on general fatigue (6 items), cognitive fatigue (6 items), and sleep/rest fatigue (6 items). The scale measures children's fatigue on a 0–100 scale, where higher scores indicate better health-related quality of life and fewer fatigue-related symptoms.

### Sleep scale

In the JIA group and the control group, children under 11 years old were assessed using the Chinese version of the Children's Sleep Habits Questionnaire (CSHQ), while those over 11 years old were evaluated using the Sleep Disturbance Scale for Children (SDSC). The CSHQ consists of 48 items (33 of which are scoring items) that assess specific sleep issues such as poor bedtime habits, sleep anxiety, irregular sleep duration, sleep breathing disorders, abnormal sleep patterns, daytime drowsiness, night awakenings, and prolonged latency to fall asleep. The CSHQ requires parents to recall their child's sleep situation over the past four weeks, selecting the most typical week (unaffected by any sudden events) and rating it on a three-point scale: “often” (5–7 times per week), “sometimes” (2–4 times per week), and “rarely” (0–1 time per week). Scoring ranges from 1 to 3, with “often” receiving 3 points, “sometimes” 2 points, and “rarely” 1 point. The two questions about daytime drowsiness are rated on a scale of “not sleepy,” “very sleepy,” or “asleep,” with scores ranging from 0 to 2. The total score is calculated by summing the scores of all 33 items. Each sub-scales score is derived from the sum of its items. Using the CSHQ, one can obtain information on children's sleep duration, the overall sleep quality score, and scores for the eight dimensions. A higher score indicates more severe sleep disorders, with 41 points being considered a clinical threshold. Based on the critical values, one can assess whether children's overall sleep quality and its various dimensions are satisfactory. The Children's Sleep Disturbance Scale (SDSC) is a widely used 26-item questionnaire. The responses on the Likert scale, ranging from 1 to 5, indicate the frequency of specific behaviors in children, with 1 indicating “never” and 5 indicating “always (every day).” The scale consists of six subscales: difficulty falling asleep and maintaining sleep, sleep breathing disorders, arousal disorders, sleep-wake transition disorders, excessive sleepiness, and night sweats. Additionally, the patient's bedtime and wake-up times are recorded. Sleep duration is determined by asking the patient about their sleep and wake-up times. The scores for each subscale are then calculated, and the total score is determined. A higher score indicates more severe sleep disorders. A SDSC score greater than 39 is considered indicative of sleep disorders.

### Statistical analysis

The analysis was conducted using the software program SPSS for Windows (version 26.0). Descriptive statistics were used to evaluate demographic variables; quantitative variables were presented as mean (Mean, M) and standard deviation (Standard Deviation, SD), or median, while qualitative variables were expressed as numbers and percentages. An independent t-test was employed to compare the CSHQ and SDSC scores between JIA patients and the control group. Spearman correlation analysis was used to determine the relationship between JIA-related variables and the total scores of CSHQ and SDSC, as well as the sub-scales scores. In all analyses, a *P*-value 0.05 was considered statistically significant.

## Results

We included 58 patients aged 4–18 years who met the inclusion criteria and were hospitalized in the Department of Pediatric Rheumatology and Immunology at Ningbo Women and Children's Hospital from June to December 2021. Patients were randomly assigned based on gender, and all patients' families agreed to participate in the study and signed informed consent forms. After inclusion, 10 patients were excluded, leaving 48 patients in the study. Additionally, 90 children with no chronic diseases who were hospitalized at the same time at Ningbo Women and Children's Hospital were selected as a control group.

The CSHQ sleep assessment scale was used to evaluate the sleep of children under 11 years old, including 20 children from the JIA group and 42 from the control group. Among the JIA group, 12 (60%) had a disease duration of less than six months, while 8 (40%) had a disease duration of more than six months. Regarding medication use: 2 (10%) were not on any medication; 15 (75%) were using one type of medication (NSAIDs); 1 (5%) were using two types of medications (NSAIDs + DMARDs); and 2 (10%) were using three or more types of medications (NSAIDs + DMARDs + biologics + others). The adverse factors for poor prognosis included 2 (10%) with adverse outcomes and 18 (90%) without. The incidence of sleep disorders was 85% in the JIA group and 57.1% in the control group. The statistical data on age and gender for both groups are shown in Table 1, with *P* > 0.05, indicating no significant difference.

**Table 1:** Demographic characteristics of patients under 11 years of age.

Characteristic	JIA group	control group	F/x^2^	*P*
	*n* = 20	*n* = 42		
Age,years	6.40 (2.30)	6.40 (1.93)	2.300	0.993
Sex (F/M)	15/5	24/18	9.336	0.163

F/M represents male/female.

The SDSC sleep assessment was used for individuals aged 11 and above, with 28 JIA patients and 48 controls included in the study. Among the JIA patients, 12 (42.9%) had a disease duration of less than six months, while 16 (57.1%) had a disease duration of more than six months. Regarding medication use: 1 person (3.6%) had no medication; 11 people (39.3%) were using one drug (NSAIDs); 4 people (14.3%) were using two drugs (NSAIDs + DMARDs); and 12 people (42.9%) were using three or more drugs (NSAIDs + DMARDs + biologics + others). In terms of adverse prognostic factors, 6 people (21.4%) had adverse outcomes, while 22 people (78.6%) did not. The incidence of sleep disorders was 28.6% in the JIA group and 10.4% in the control group. The statistical data on age and gender for both groups are shown in Table 2, with *P* > 0.05, indicating no significant difference.

**Table 2:** Demographic characteristics of patients over 11 years of age.

Characteristic	JIA group	control group	F/x^2^	*P*
*n*	28	48	–	–
Age (years)	12.71 (1.301)	12.65 (1.246)	0.147	0.821
Sex (F/M)	21/7	24/24	15.579	0.087

### Analysis of JIA related variables

The results of JIA-related variables such as disease activity, functional status, pain, and fatigue in children under 11 years old are shown in Table 3.

**Table 3:** JIA related variables.

Characteristic	M (SD)	Median
ESR (mean SD)	0.055 (0.246)	0.000
PGE (cm)	3.660 (1.817)	3.500
PGA (cm)	3.235 (1.794)	2.700
AJC	1.75 (1.209)	1.00
JADAS-27	8.700 (3.687)	7.650
PedsQLTM MFS	1,478.75 (246.859)	1,512.50
CHQ-PF28	19.65 (3.951)	19.50
VAS (cm)/Wong-Baker	4.180 (2.116)	4.600

ESR, stands for erythrocyte sedimentation rate; PGE, stands for parent overall evaluation; PGA, stands for physician global assessment; AJC, stands for active joint count; JADAS-27, stands for Juvenile Arthritis Disease Activity Score 27; PedsQLTM MFS, stands for PedsQLTM Multidimensional Fatigue Scale for Children; CHQ-PF28, stands for Health Questionnaire for Children with Idiopathic Arthritis; VAS/Wong-Baker, visual analog scale or facial expression pain intensity rating.

The results of JIA-related variables such as disease activity, functional status, pain, and fatigue in patients over 11 years of age are shown in Table 4.

**Table 4:** JIA related variables.

Instrument	M (SD)	Median
ESR (mean SD)	0.657 (1.773)	0.000
PGE (cm)	3.625 (1.834)	3.050
PGA (cm)	3.196 (1.444)	3.050
AJC	2.36 (2.614)	2.00
JADAS-27	9.836 (5.705)	8.450
PedsQLTM MFS	1,442.86 (232.723)	1,425.00
CHQ-PF28	19.75 (4.477)	19.00
VAS (cm)	4.093 (2.694)	4.300

### CSHQ sleep analysis was performed in JIA group and control group

The CSHQ sleep scores of the JIA group and the control group are shown in Table 5. Independent t-tests revealed that the total CSHQ score of JIA patients was significantly higher than that of the control group (*p* = 0.000). Significant differences were also observed across multiple subscales, with a highly significant difference in sleep duration between the JIA group and the control group (*p* = 0.000), as well as significant differences in sleep anxiety (*p* = 0.046), night awakenings (*p* = 0.002), abnormal sleep patterns (*p* = 0.003), sleep breathing disorders (*p* = 0.000), and daytime sleepiness (*p* = 0.007).

**Table 5:** CSHQ sleep scores of JIA group and control group.

Variable	JIA M (SD)	control M (SD)	*P*
CSHQ score	49.90 (7.130)	41.88 (7.188)	0.000
Bedtime resistance	11.10 (2.936)	10.26 (3.254)	0.332
Sleep-onset delay	1.60 (0.821)	1.50 (0.741)	0.633
Sleep duration	3.25 (0.639)	2.02 (0.154)	0.000
Sleep anxiety	7.00 (2.200)	5.81 (2.121)	0.046
Night wakings	4.75 (1.650)	3.62 (1.125)	0.002
Parasomnias	8.15 (1.309)	7.14 (1.160)	0.003
Sleep-disordered breathing	3.10 (0.308)	2.24 (0.576)	0.000
Daytime sleepiness	10.95 (2.282)	9.29 (2.415)	0.007

In the JIA group, there was a significant correlation between the total CSHQ score and PGA (*p* = 0.021), AJC (*p* = 0.041), and JADAS-27 scores (*p* = 0.05). Sleep duration was associated with the standardized ESR value (*p* = 0.002), and bedtime habits were linked to AJC (*p* = 0.045). Daytime sleepiness was correlated with PGE (*p* = 0.017), PGA (*p* = 0.022), and JADAS (*p* = 0.029). The latency to fall asleep was related to the Multidimensional Fatigue Scale (MDS) score (*p* = 0.014), and sleep duration was associated with the CHQ-PF28 score (*p* = 0.006). The overall CSHQ or CSHQ subscale scores were not directly related to pain, medication use, or adverse factors of disease prognosis (Table 6.).

**Table 6:** Correlation between JIA-related variables and sleep disorders in patients under 11 years of age.

Characteristic	CSHQ score	Bedtime resistance	Sleep-onset delay	Sleep duration
ESR (mean)	0.771	0.973	0.468	0.002
PGE (cm)	0.569	0.685	0.364	0.166
PGA (cm)	0.021	0.875	0.139	0.133
AJC	0.041	0.045	0.369	0.943
JADAS-27	0.05	0.363	0.419	0.233
PedsQLTM MFS	0.699	0.349	0.014	0.132
CHQ-PF28	0.942	0.329	0.693	0.006
VAS/Wong-Baker	0.133	0.628	0.625	0.845
prognostic factors	0.702	0.770	0.862	0.574
Medicine	0.486	0.205	0.665	0.728

Characteristic	Sleep anxiety	Night wakings	Parasomnias	Sleep-disordered breathing	Daytime sleepiness
ESR	0.654	0.881	0.910	0.749	0.371
PGE (cm)	0.800	1.000	0.782	0.670	0.017
PGA (cm)	0.867	0.454	1.000	0.946	0.022
AJC	0.084	0.102	0.862	0.767	0.674
JADAS-27	0.595	0.885	0.841	0.954	0.029
PedsQLTM MFS	0.135	0.534	0.164	0.983	0.154
CHQ-PF28	0.475	0.198	0.467	0.548	0.190
VAS/Wong-Baker	0.321	0.247	0.567	0.903	0.070
Prognostic factors	0.513	0.270	0.127	0.641	0.111
Medicine	0.217	0.621	0.418	0.773	0.811

### SDSC sleep analysis was performed in JIA group and control group

The SDSC sleep scores of the JIA group and the control group are shown in Table 7. Independent *t*-tests revealed that the total SDSC score of JIA patients was significantly higher than that of the control group (*p* = 0.000). Significant differences were also observed across multiple subscales, with JIA children showing a highly significant difference from the control group in wakefulness disorders (*p* = 0.000), sleep breathing disorders (*p* = 0.001), sleep-wake transition disorders (*p* = 0.021), and excessive sleepiness (*p* = 0.015).

**Table 7:** SDSC sleep scores of JIA group and control group.

Instrument	JIA group M (SD)	control M (SD)	*P*
SDSC score	36.82 (6.815)	30.44 (7.305)	0.000
DIMS^a^	11.75 (3.384)	10.31 (3.725)	0.098
Sleep Breathing Disorders	3.36 (0.559)	2.69 (1.055)	0.001
Disorders of Arousal	3.07 (0.262)	2.19 (0 0.532)	0.000
Parasomnias	8.86 (2.520)	7.50 (2.370)	0.021
Hypersomnia	7.25 (2.398)	5.71 (2.729)	0.015
Sleep hyperhidrosis	2.54 (1.319)	2.04 (1.738)	0.197

a DIMS, Disorders of Initiating and Maintaining Sleep.

In the JIA group, there was a significant correlation between SDSC night sweats and PGA (*p* = 0.039), AJC (*p* = 0.038), and JADAS-27 scores (*p* = 0.032). Excessive sleepiness was associated with the Multidimensional Fatigue Scale (*p* = 0.014) and CHQ-PF28 scores (*p* = 0.038). Sleep disorders (*p* = 0.029) and night sweats (*p* = 0.046) were linked to medication use. The overall SDSC or SDSC subscale scores were not directly related to pain or poor disease prognosis factors (Table 8).

**Table 8:** Correlation between JIA related variables and sleep disorders.

Characteristic	SDSC score	DIMS Sleep	Breathing Disorders
	*p*	*p*	*p*
ESR	0.849	0.503	0.374
PGE (cm)	0.181	0.209	0.382
PGA (cm)	0.212	0.602	0.461
AJC	0.543	0.425	0.840
JADAS-27	0.357	0.967	0.403
PedsQLTM MFS	0.067	0.399	0.861
CHQ-PF28	0.144	0.755	0.793
VAS (cm)	0.107	0.189	0.386
Prognostic factors	0.602	0.643	0.356
Medicine	0.952	0.989	0.215

Characteristic	Disorders of Arousal	Parasomnias	Hypersomnia	Sleep hyperhidrosis
	*p*	*p*	*p*	*p*
ESR (mean)	0.596	0.691	0.919	0.066
PGE (cm)	0.984	0.479	0.182	0.791
PGA (cm)	0.924	0.540	0.443	0.039
AJC	0.845	0.640	0.176	0.038
JADAS-27	0.776	0.637	0.202	0.032
PedsQLTM MFS	0.965	0.580	0.014	0.059
CHQ-PF28	0.690	0.054	0.038	0.287
VAS (cm)	0.215	0.452	0.161	0.312
Prognostic factors	0.325	0.459	0.779	0.543
Medicine	0.029	0.663	0.243	0.046

## Discussion

Sleep, which makes up one-third of a person's life, is an essential component of human health. Adequate sleep is crucial for the physical and mental well-being of children, as well as for their neurological and behavioral functions and regulation (9, 10). A study involving 998 children and adolescents aged 12–17 found that habitual shortening of nighttime sleep is a common issue among children and adolescents, with significantly higher rates of insomnia, delayed sleep phase syndrome, restless leg syndrome, and obstructive sleep apnea (11). Research on adolescents with JIA abroad has shown that the prevalence of sleep disorders is high, ranging from 20% to 70% based on the CSHQ (12). The most common reported sleep issues include delayed sleep onset, wakefulness after sleep, abnormal sleep patterns, anxiety, sleep breathing disorders, and insufficient sleep duration (13). In this study, the prevalence of sleep disorders assessed by the CSHQ among JIA patients under 11 years old was 85%, and among those over 11 years old, the prevalence was 28.6%. The total score for sleep disorders was significantly higher than in the general population in China. Our research also found that JIA children had significantly higher scores for sleep disorders compared to healthy children. Significant differences were observed in sleep duration (*p* = 0.000), sleep anxiety (*p* = 0.046), night awakenings (*p* = 0.002), heterogeneous sleep (*p* = 0.003), sleep breathing disorders (*p* = 0.000), and daytime sleepiness (*p* = 0.007). Additionally, there were significant differences in wakefulness disorders (*p* = 0.000), sleep breathing disorders (*p* = 0.001), sleep-wake transition disorders (*p* = 0.021), and excessive sleepiness (*p* = 0.015), consistent with the findings of Ward Teresa M (14) et al. However, some studies have found no significant differences in total sleep time, post-sleep awakenings, or sleep efficiency using PSG monitoring (15). Regarding the mechanisms of sleep disorders in JIA children, previous research has suggested that JIA patients exhibit a significant inflammatory response, including a decrease in IL-10 and an increase in pro-inflammatory cytokines. A recent study on JIA found that chronic inflammatory signaling, primarily through the upregulation of TNF-α in the indoleamine-2,3-dioxygenase pathway, leads to an increased ratio of tryptophan to uracil and increased brain breakdown. This reduces the synthesis of serotonin in the nucleus accumbens and melatonin in the pineal gland, potentially disrupting circadian rhythms and impairing sleep (16).

Research on JIA sleep disorders is still in its early stages. In this study, gender, disease activity, functional status, and fatigue are associated with sleep disorders. Male patients in the JIA group are significantly more common than female patients, possibly due to social factors and family's willingness to seek medical care. Additionally, the study found that as the number of medications used by older JIA children increases compared to younger JIA children, adverse prognostic factors also increase. Our research found a significant correlation between the total score of CSHQ and PGA (*p* = 0.021), AJC (*p* = 0.041), and JADAS-27 score (*p* = 0.05). Sleep duration is correlated with ESR standardized values (*p* = 0.002), bedtime habits are related to AJC (*p* = 0.045), and daytime drowsiness is associated with PGE (*p* = 0.017), PGA (*p* = 0.022), and JADAS (*p* = 0.029). In the SDSC, night sweats in the SDSC are significantly correlated with PGA (*p* = 0.039), AJC (*p* = 0.038), and JADAS-27 score (*p* = 0.032), indicating a significant relationship between sleep disorders and disease activity, consistent with the findings of Shyen, S et al. (12). Georgia Tsipoura et al. (17) found that non-active JIA children have higher CSHQ scores and increased sleep time, but they believe that the sleep disorder index is not related to JIA disease activity. This study also found a correlation between sleep and functional status. In the CSHQ, sleep duration was associated with functional status (*p* = 0.006), and in the SDSC, excessive sleepiness was linked to functional status (*p* = 0.038). These findings suggest that these factors may be related to the mental state, physical condition, social engagement, and family support of the children. Additionally, the study found that the social environment is closely linked to sleep. Recent studies indicate that during the COVID-19 pandemic, the prevalence of sleep problems among adolescents and young adults is high (18). Furthermore, 14.7% of newly diagnosed JIA adolescents experienced high levels of depressive symptoms (19). Insufficient sleep and psychological distress (such as depression, anxiety, and stress) are closely related and mutually influence each other (20). Sleep itself can regulate emotions and motivation (21, 22), and insufficient sleep can directly impact psychological well-being, including increasing the severity of depression, anxiety, and stress.

Fatigue is a symptom resulting from the complex interplay of various factors, a disabling symptom of multiple chronic diseases, and a common complaint in JIA. According to reports, 60%–76% of JIA patients experience fatigue, which can significantly impact daily life, leading to poor sleep quality and daytime drowsiness. A recent prospective cohort study in Northern Europe, which followed 377 JIA children for 18 years, found that 26% of JIA patients reported severe fatigue. JIA-related fatigue is closely linked to sleep disorders, physical disability, and disease activity (23). Tarakci E et al. (24) found that 75% of JIA patients experienced moderate to high levels of fatigue, with sleep duration being the strongest predictor of the severity of fatigue. The severity of fatigue, sleep quality, and functional ability are also predictive factors for fatigue symptoms. This study found that fatigue scores are closely related to sleep disorders, with the CSHQ's sleep latency (*p* = 0.014) and SDSC's excessive sleepiness (*p* = 0.014) both associated with fatigue, consistent with previous findings,suggesting that interventions targeting sleep quality may break the vicious cycle of fatigue-sleep disturbance and improve daily functioning.

Research has found that children with chronic pain have pain symptoms and subjective reports of sleep disorders (25–31). Yonatan Butbul Aviel et al. (32) found that nearly half of patients with JIA and juvenile dermatomyositis experience disrupted sleep, and there is a significant relationship between sleep disorders, fatigue, pain, disease activity, and health-related quality of life. Pain can affect sleep quality, which in turn can influence how children perceive and respond to pain, potentially leading to a vicious cycle. Bromberg, Maggie H et al. (33) used a prospective study method and found that sleep quality and difficulty falling asleep are associated with the intensity of pain, but there is little correlation between sleep duration and pain. However, our study found that the total scores of the CSHQ or its sub-scales, and the total scores of the SDSC or its sub-scales, were not directly related to pain. This may be because most of our sample was over 11 years old, and these children often use medication to manage their conditions, resulting in lower pain scores.

In the study on the correlation between JIA treatment drugs and sleep, we found that in the JIA group aged 11 and above with a high proportion of three or more medications (including biologics), the occurrence of wakefulness disorders (*p* = 0.029) and night sweats (*p* = 0.046) is associated with medication use. Previous studies have shown While TNF-α inhibitors have shown potential to reduce inflammation-related sleep disturbances in some studies, the evidence for their efficacy in improving obstructive sleep apnea (OSA) remains limited, and further research is needed to confirm these effects (34, 35), possibly due to their ability to reduce inflammation. Additionally, research indicates that anti-rheumatic drugs or biologics do not significantly affect sleep (19), but there are also reports suggesting that glucocorticoids can make it difficult to initiate and maintain sleep, and non-steroidal anti-inflammatory drugs may have potential negative effects on sleep. Therefore, the impact of these drugs on sleep still requires further investigation.

Overall, given the high incidence and adverse effects of sleep disorders (36, 37), routine sleep screening using validated tools such as CSHQ and SDSC should be integrated into standard care for JIA patients to enable early identification and intervention, thereby improving quality of life. Our assessment provides a basis for recognizing the importance of sleep disorders in JIA children. The evaluation of sleep disorders in school-age children can be conducted through clinical interviews with parents and children, as well as using screening tools like CSHQ. For adolescents and children, sleep disorders can be assessed using scales such as SDSC (38), which provide valuable insights into sleep duration and habits. Additionally, treating sleep disorders requires a multifaceted approach, focusing on supporting JIA children by improving their family environment, social interactions with peers, academic performance, and psychological well-being, to ensure they have positive emotions that facilitate learning and daily life (39–41). Moreover, it is crucial to consider the medication regimen of JIA patients. However, our study has certain limitations. Currently, most studies use subjective self-assessment scales to evaluate sleep quality,which may overestimate or underestimate sleep disturbances compared to objective measures like polysomnography (PSG). Future studies should incorporate PSG where feasible. However, due to the need for hospitalization, its implementation in children is currently challenging, and whether changes in the sleep environment affect the results remains unclear. Additionally, our study had a limited scope, a small sample size, which may reduce statistical power to detect significant differences in subdomains such as sleep hyperhidrosis (*p* = 0.197 in Table 8). Caution is needed when interpreting non-significant findings, and no longitudinal observation, which may introduce biases. A larger-scale study is needed to make these findings more applicable and generalizable to other populations.

In summary, children with JIA (Juvenile Arthritis) suffer from sleep disorders, which are significantly disrupted. The disease's activity, fatigue, functional status, and medication use are closely linked to these sleep disorders, leading to poorer sleep quality and negatively impacting the child's quality of life. While focusing on the disease itself, clinicians should also pay attention to the patient's sleep conditions and related factors, aiming to implement early and proactive sleep interventions to enhance the patient's overall quality of life, increase their satisfaction with life, and improve the disease's prognosis.

## Data Availability

The original contributions presented in the study are included in the article/Supplementary Material, further inquiries can be directed to the corresponding author.
